# Versatile Roles of the Chromatin Remodeler CHD7 during Brain Development and Disease

**DOI:** 10.3389/fnmol.2017.00309

**Published:** 2017-09-28

**Authors:** Weijun Feng, Chunxuan Shao, Hai-Kun Liu

**Affiliations:** Division of Molecular Neurogenetics, German Cancer Research Center, DKFZ-ZMBH Alliance, Heidelberg, Germany

**Keywords:** CHD7, chromatin remodeler, CHARGE syndrome, brain development, mouse models

## Abstract

CHD7 (Chromo-Helicase-DNA binding protein 7) protein is an ATP-dependent chromatin remodeler. Heterozygous mutation of the *CHD7* gene causes a severe congenital disease known as CHARGE syndrome. Most CHARGE syndrome patients have brain structural anomalies, implicating an important role of CHD7 during brain development. In this review, we summarize studies dissecting developmental functions of CHD7 in the brain and discuss pathogenic mechanisms behind neurodevelopmental defects caused by mutation of *CHD7*. As we discussed, CHD7 protein exhibits a remarkably specific and dynamic expression pattern in the brain. Studies in human and animal models have revealed that CHD7 is involved in multiple developmental lineages and processes in the brain. Mechanistically, CHD7 is essential for neural differentiation due to its transcriptional regulation in progenitor cells.

## Heterozygous Mutation of *CHD7* Leads to Brain Developmental Anomalies

The human *CHD7* (Chromo-Helicase-DNA binding protein 7) is a long gene spanning approximately 189 kb at chromosome 8, containing 38 exons encoded for a large protein (2997 aa, about 336 kD). CHD7 protein is a member of CHD family of ATP-dependent chromatin remodelers. These enzymes utilize the energy from ATP hydrolysis to mobilize or relocate nucleosomes, thereby control DNA accessibility of chromatin. Chromatin remodeling is crucial for DNA-related biological processes such as transcription, chromosome segregation, DNA replication, and DNA repair (Clapier and Cairns, 2009). Not surprisingly, most chromatin remodelers are indispensable for normal development (Ho and Crabtree, 2010).

CHARGE syndrome (OMIM #214800), initially described in 1979 (Hall, 1979; Hittner et al., 1979) and named in 1981 (Pagon et al., 1981), is a congenital disease with severe developmental defects in multiple organ systems. Two decades later, *de novo* mutations in the *CHD7* gene were identified in CHARGE syndrome patients (Vissers et al., 2004), which turn out to be the major cause of this disease. Over 90% of patients with clinically typical CHARGE syndrome have heterozygous mutations in the *CHD7* gene (Bergman et al., 2011). Moreover, mutations of *CHD7* have also been identified in about 6% of Kallmann syndrome (OMIM #308700), a developmental disease characterized with IHH (idiopathic hypogonadotropic hypogonadism) and anosmia (Kim et al., 2008; Balasubramanian et al., 2014; Marcos et al., 2014). Up to now, 554 pathogenic *CHD7* mutations have been identified in CHARGE syndrome patients.^1^ About 90% of these mutations are nonsense, frame shift, and splice site mutations, which result in truncated CHD7 protein (Basson and van Ravenswaaij-Arts, 2015). In contrast, more than 70% of *CHD7* mutations in Kallmann syndrome are missense mutations (Balasubramanian et al., 2014; Marcos et al., 2014), which correlate with mild phenotypes in these patients as compared to CHARGE syndrome.

Several earlier review articles have comprehensive summarized the developmental roles of CHD7 in multiple organs affected in CHARGE syndrome patients (Layman et al., 2010; Zentner et al., 2010; Bergman et al., 2011). This review focuses on the function of CHD7 during brain development. Multiple structural defects in the brain of CHARGE syndrome patients have been reported, such as hypoplasia of olfactory bulb and cerebellum, agenesis of the corpus callosum, microcephaly and atrophy of the cerebral cortex (Lin et al., 1990; Tellier et al., 1998; Becker et al., 2001; Johansson et al., 2006; Sanlaville et al., 2006; Legendre et al., 2012; Yu et al., 2013; Hale et al., 2016). Among them, deficiency of olfactory bulb and sulci is the most frequent brain defect in CHARGE syndrome patients, as shown by Magnetic Resonance Imaging (MRI) (Chalouhi et al., 2005; Pinto et al., 2005; Blustajn et al., 2008). Because only small cohorts of patients were being examined in these studies and the phenotype of CHARGE syndrome is very heterogeneous, the overall percentage of patients having brain developmental anomalies is still not known. Nevertheless, brain structural defect has recently been proposed as minor criteria for the diagnosis of CHARGE syndrome (Hale et al., 2016). Consistent with defects in the brain, most CHARGE syndrome patients have certain degree of intellectual deficiency (Bergman et al., 2011). In Kallmann syndrome patients with *CHD7* mutations, the common brain phenotype is hypoplasia of olfactory bulb and reduced number of GnRH (gonadotropin-releasing hormone) neurons in the hypothalamus (Marcos et al., 2014). Together, these observations clearly demonstrate that CHD7 is haploinsufficient for brain development.

## The Expression of *CHD7* in the Brain is Very Specific and Dynamic

**FIGURE 1:**
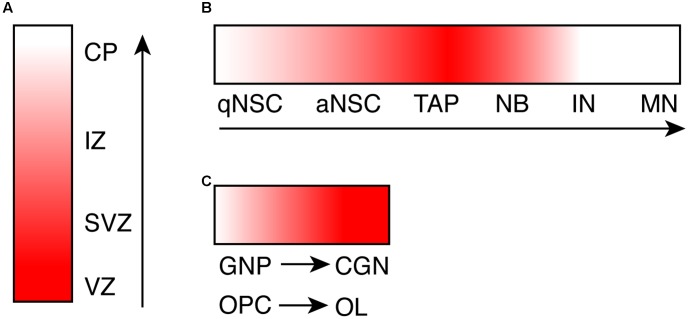
The dynamic expression pattern of CHD7 during neurogenesis and oligodendrogenesis. **(A)** A cartoon shows the expression level of *Chd7* transcript during embryonic neurogenesis, based on *in situ* hybridization data. **(B)** A cartoon shows the expression level of CHD7 protein in adult neurogenic lineage in hippocampal dentate gyrus, based on immunostaining results. **(C)** A cartoon shows the increase of CHD7 expression upon differentiation of GNP and OPC, based on immunostaining results. The degree of redness reflects the expression level of *Chd7*. Arrows show the direction of differentiation. VZ, ventricular zone; SVZ, subventricular zone; IZ, intermediate zone; CP, cortical plate; qNSC, quiescent neural stem cell; aNSC, active NSC; TAP, transit amplifying progenitor; NB, neuroblast; IN, immature neuron; MN, mature neuron; GNP, granule neuron progenitor; CGN, cerebellar granule neuron; OPC, oligodendrocyte precursor cell; OL, oligodendrocyte.

Consistent with specific brain defects caused by *CHD7* mutations, the *CHD7* gene exhibits a spatial- and temporal-specific expression pattern during brain development. Expression of *CHD7* in the human brain has been observed throughout development (Sanlaville et al., 2006). More detailed analysis of the expression of *Chd7* has been done in mouse brain. Using *in situ* hybridization analysis, one study demonstrates that *Chd7* is expressed as early as E8.5 (Embryonic day 8.5) in mouse brain regions including forebrain and midbrain neural fold, neural tube, and neuroepithelial prominence (Jiang et al., 2012). At E12.5 and E14.5, *Chd7* is highly expressed in frontal cortex, medial ganglionic eminence, ventricular zone of medulla, and external granule zone of cerebellum (Bosman et al., 2005). A similar expression pattern of *Chd7* was observed in *Chd7^Gt/+^* (Gt: Gene-trap) embryo, where the expression of β-galactosidase reporter closely mimics endogenous *Chd7* (Hurd et al., 2007). One common observation from these studies is that the expression level of *Chd7* is higher in the proliferating ventricular zone compared to the differentiated areas of the neuroepithelium (**Figure 1A**). In adult mouse brain, *Chd7* shows a very specific and dynamic expression pattern in the adult neurogenic region subgranular zone (SGZ) in hippocampal dentate gyrus (DG) (**Figure 1B**). With immunostaining analysis, CHD7 is found to be expressed upon the activation of adult neural stem cells (NSCs), and is upregulated in transit progenitor cells and neuroblasts. In contrast, the expression of *Chd7* is completely switched off in granule neurons in DG (Feng et al., 2013; Jones et al., 2015). This dynamic expression pattern of *Chd7* in DG was confirmed in a recent single-cell RNA sequencing study whereas transcriptomes of EdU pulse-labeled individual neuronal cells were analyzed (Habib et al., 2016). Consistent with *in vivo* data showing the upregulation of *Chd7* upon activation of adult NSCs, *Chd7* is downregulated in cultured NSCs upon BMP4-induced quiescence (Martynoga et al., 2013).

In cerebellum, immunostaining results show that CHD7 is highly expressed in cerebellar granule cells throughout development and persists in adult cerebellum, in contrast to almost no expression in Purkinje neurons and Bergmann glia cells (Feng et al., 2017). In contrast to granule neurons in DG, the expression level of Chd7 is even higher in differentiated cerebellar granule neurons compared to granule neuron progenitors (GNPs) (Feng et al., 2017) (**Figure 1C**). Besides neuronal cells in the brain, while Chd7 is barely expressed in astrocytes, Chd7 is broadly expressed in oligodendrocyte (OL) lineage (He et al., 2016). Similar to cerebellar granule cells, the expression level of *Chd7* in differentiated oligodendrocytes is higher than oligodendrocyte precursor cells (OPCs) (**Figure 1C**). With such a specific and dynamic expression pattern in the brain, the expression of *Chd7* is expected to be tightly regulated. Mechanism behind is currently not known, however.

## Loss-of-Function Study in Mouse Model Reveals An Important Role of *CHD7* During Brain Development

While xenopus (Bajpai et al., 2010), drosophila (Melicharek et al., 2010), and zebrafish models (Patten et al., 2012) have been established, mouse model is currently the predominant animal model for functional study of CHD7 and CHARGE syndrome. As shown in CHARGE syndrome patients, data from mouse studies confirm that CHD7 is haploidinsufficient for brain development (**Table 1**). Mice carrying germline loss-of-function mutation of *Chd7* have been generated with both ethylnitrosourea (ENU)-induced mutations and the gene-trap approaches. While homozygous loss-of-function mutation of *Chd7* is embryonic lethal at E10.5, heterozygous mutant mice are viable but show phenotypes closely mimicking CHARGE syndrome (Bosman et al., 2005; Hurd et al., 2007). Analysis of E10.5 *Chd7* homozygous gene-trap mutant embryo shows a reduction of the thickness of neuroepithelium in telencephalon and midbrain (Hurd et al., 2007), indicating that *Chd7* is required for early brain development. Heterozygous *Chd7* loss-of-function mutant mice show defects in different brain regions. The most frequent defect is the absence or hypoplasia of olfactory bulb (Layman et al., 2009; Bergman et al., 2010; Jiang et al., 2012). Other brain anomalies identified in these mice include reduced number of GnRH neurons in the hypothalamus (Layman et al., 2011), defects in the development of telencephalic midline and reduction of the thickness of cerebral cortex (Jiang et al., 2012).

**Table 1:** Brain phenotype in *Chd7* loss-of-function mutant mouse models.

Mouse lines	Mouse brain phenotype	Relevant CHARGE phenotype	Reference
*Chd7^Gt(S20-7E1)/Gt(S20-7E1)^* (Gene-trap reporter inserted in exon 1)	E10.5 lethal, hypoplasia of the neuroepithelium	Cognitive disability	Hurd et al., 2007
*Chd7^Gt(S20-7E1)/+^*	Small olfactory bulb (OB)	Hyposmia	Layman et al., 2009
*Chd7^Whi/+^* (p.W973X) *Chd7^Gt(S20-7E1)/+^*	OB hypoplasia; decreased GnRH neurons in the hypothalamus	Anosmia; Genital hypoplasia; Puberty delay	Bergman et al., 2010; Layman et al., 2011
*Chd7^COA1/+^* (p.K719X)	Hypoplasia of OB; Telencephalic midline defects; Reduced cerebral cortex	Hyposmia; Cognitive disability	Jiang et al., 2012
*Nestin-CreERT2::Chd7 ^f/f^ Ubc-CreERT2::Chd7 ^f/f^ Glast-CreERT2::Chd7 ^f/f^*	Defects of adult neurogenesis	Cognitive disability; Hyposmia	Feng et al., 2013; Micucci et al., 2014; Jones et al., 2015
*Chd7^Gt(XK403)/+^* (Gene-trap reporter inserted in intron 36) *Atoh1-Cre::Chd7^f/f^ Nestin-Cre::Chd7^f/f^*	Vermis hypoplasia; Purkinje cell heterotopia	Vermis hypoplasia; Purkinje cell heterotopia	Yu et al., 2013; Feng et al., 2017; Whittaker et al., 2017
*Olig1-Cre::Chd7 ^f/f^ Pdgfr-CreERT2::Chd7 ^f/f^*	Defect of myelination	White matter defects	He et al., 2016


Ablation of *Chd7* in germline is embryonic lethal at E10.5, which prevents the study of its function in brain development during later stage. In order to further dissect the function of *Chd7* in brain development, several labs have applied *Chd7* conditional knockout (CKO) mouse lines (*Chd7^flox/flox^*) (**Table 1**). Two *Chd7^flox/flox^* mouse lines have been used, with either exon 2 or 3 being floxed (Hurd et al., 2010; Feng et al., 2013). Upon cre-mediated recombination, the CHD7 protein is ablated in CKO mouse. Compared to mice carrying germline loss-of-function mutation of *Chd7*, key advantage of *Chd7* CKO mouse line is that it allows researchers to dissect the function of *Chd7* in specific cell lineage during brain development in a spatial- and temporal-specific manner. As discussed below, crucial functions of *Chd7* in adult neurogenesis, cerebellar development, and CNS myelination have been revealed by the CKO approach.

Adult neurogenesis occurs in restricted mouse brain regions including SGZ of DG in hippocampus and subventricular zone (SVZ) of lateral ventricle (Gage, 2000). Given neurogenic mechanism in adult is similar to embryonic stage, adult neurogenesis provides an ideal system to study neurodevelopment. Moreover, while germline homozygous loss-of-function mutations of many neurodevelopmental genes are embryonic lethal, adult neurogenesis, in the other hand, allows the study of homozygous gene-silencing mutations because majority of them do not affect animal survival. Three independent studies demonstrated that loss of *Chd7* in adult NSCs leads to a decline of adult neurogenesis (Feng et al., 2013; Micucci et al., 2014; Jones et al., 2015). In *Chd7* CKO brain, the number of newborn neurons reduces, and mutant neurons show clear defects in dendritic development. Strikingly, voluntary running, a positive stimulus of adult neurogenesis in hippocampus, could rescue hippocampal neurogenic defects, including both the amount and the dendritic development of newborn neurons in *Chd7* CKO mice (Feng et al., 2013). Mutation of *Chd7* in adult NSCs was also shown to result in a loss of quiescent stem cells in DG, thereby an exhaustion of NSC pool (Jones et al., 2015). Dissecting mechanism behind the adult neurogenic defect in *Chd7* CKO mice reveals the function of CHD7 in neurodevelopment, which may help us to understand the cognitive deficiency that is frequently observed in CHARGE syndrome patients.

Structural defects in cerebellum occur frequently in CHARGE syndrome patients (Becker et al., 2001; Sanlaville et al., 2006; Legendre et al., 2012; Yu et al., 2013). Recent mouse studies have provided valuable insights into mechanism behind the pathogenesis of cerebellar anomalies caused by loss-of-function of *Chd7*. Heterozygous gene-trap mutation of *Chd7* leads to reduced expression of key signaling molecule Fgf8 (Fibroblast growth factor 8) in isthmus organizer (IsO), an organizer region that directs cerebellar development during early embryonic stage (between E8 and E9). Importantly, heterozygous loss-of-function mutations of both *Chd7* and *Fgf8* show a synergistic effect in cerebellar development, resulting in severe vermis aplasia (Yu et al., 2013). While this study provides evidence showing the important role of *Chd7* during early embryonic development of cerebellum, recent studies have revealed that *Chd7* is also required for cerebellar development at later stages. Using the *Atoh1-Cre::Chd7 ^flox/flox^* mouse line to knockout *Chd7* specifically in GNPs, two independent studies clearly reveal that *Chd7* mutant animals exhibit cerebellar hypoplasia and massive Purkinje cell heterotopia (Feng et al., 2017; Whittaker et al., 2017). Importantly, highly similar phenotypes were observed in CHARGE syndrome pre-fetuses and patients (Legendre et al., 2012; Yu et al., 2013). Ablation of *Chd7* in Purkinje cell progenitors in the *Ptf1a-Cre::Chd7^flox/flox^* mice does not result in any obvious cerebellar developmental defect (Feng et al., 2017), excluding an essential role of *Chd7* in Purkinje cell lineage. Together, these findings strongly implicate that dysfunction of CHD7 in cerebellar granule cell lineage leads to cerebellar defects in CHARGE patients.

Consistent with the specific expression of *Chd7* in OL lineage, CHD7 has been shown to play a crucial role in myelination during brain development and remyelination after drug-induced demyelination in adult mice (He et al., 2016). Further mechanistic study show that *Chd7* is required for differentiation and maturation of OLs. The function of CHD7 in oligodendrogenesis may help to explain the structural defects in white matter and corpus callosum of CHARGE syndrome patients.

## Loss of *CHD7* in the Mouse Brain Causes Common Cellular Phenotype

As discussed above, CHD7 is involved in both neurogenesis and oligodendrogenesis, highlighting its versatile roles during brain development. On the other hand, loss of *Chd7* in different cell lineages in mouse brain causes similar cellular phenotype. **First**, loss of *Chd7* leads to defect in terminal differentiation of mouse neural progenitor cells. Ablation of *Chd7* in mouse adult NSCs or cerebellar GNPs impairs terminal differentiation of granule neurons in DG and cerebellum, respectively (Feng et al., 2013, 2017). Similarly, ablation of *Chd7* in mouse OPCs leads to defects in the differentiation of OLs (He et al., 2016). **Second**, Chd7 seems to be dispensable for both the generation and the proliferation of neural progenitor cells. Actually, ablation of *Chd7* in adult NSCs in both *Nestin-CreERT2::Chd7^flox/flox^* and *Glast-CreERT2::Chd7^flox/flox^* mice leads to a mild increase of cell proliferation in the SGZ (Feng et al., 2013; Jones et al., 2015). The specification and proliferation of cerebellar GNPs are not affected in *Atoh1-Cre::Chd7^flox/flox^* mice (Feng et al., 2017). Knockout of *Chd7* in OL lineage in *Olig1-Cre::Chd7^flox/flox^* mice, does not affect the generation and proliferation of OPCs (He et al., 2016). **Third**, loss of *Chd7* in neural progenitor cells leads to increased cell death. Increased cell death was observed upon loss of *Chd7* in both adult neurogenic regions and cerebellum in mouse (Feng et al., 2013, 2017; Whittaker et al., 2017). Cultured *Chd7* mutant mouse GNPs are more prone to cell death upon differentiation (Feng et al., 2017). Interestingly, loss of *Chd7* was shown to activate p53-dependent induction of apoptosis during embryonic development, and inhibition of p53-dependent apoptosis could partially rescue developmental defects in *Chd7* knockout embryo (Van Nostrand et al., 2014). Whether p53-dependent activation of cell death occurs during adult neurogenesis and cerebellar development need to be tested. In summary, CHD7 seems to have a similar cellular function in different cell lineages during brain development. CHD7 is essential for both neurogenesis and oligodendrogenesis via controlling the differentiation of neural progenitor cells. Loss of *Chd7* in progenitor cells has mild effect on cell proliferation, but leads to the increase of cell death.

## Key Target Genes of CHD7 During Brain Development

Consistent with the known function of chromatin remodelers in transcriptional regulation, a common molecular function of CHD7 in neural cells is controlling gene expression. Multiple target genes of CHD7 during brain development have been identified (**Table 2**). Using shRNA-mediated gene knock down approach in cultured mouse NSCs, Engelen et al. (2011) show that CHD7 functions as a cofactor of the SoxB family of transcription factor SOX2 to activate multiple developmental disease-relevant genes like *Jag1*, *Gli2*, *Gli3*, and *MycN*. In adult mouse NSCs, CHD7 is required for the activation of two SoxC family of transcription factors SOX4 and SOX11, and overexpression of SOX4 and SOX11 could largely rescue the neuronal differentiation defect in *Chd7* mutant NSCs (Feng et al., 2013). Another study show that CHD7 activates the expression RA (retinoic acid) signaling receptors *Rarb* and *Rxrg*, and RA treatment could partially rescue neuronal differentiation defect in neurosphere derived from the SVZ of *Chd7* CKO mice (Micucci et al., 2014). Also, CHD7 was shown to activate Notch effector HES5 in quiescent adult mouse NSCs, which is known to be required for the maintenance of NSC quiescence (Jones et al., 2015). During early cerebellar development, CHD7 was found to activate and repress the expression of *Gbx2* and *Otx2*, respectively, which results in the downregulation of *Fgf8* in IsO (Yu et al., 2013). During postnatal development of cerebellum, two independent studies reported *reelin*, an essential gene for neuron migration, as a key target gene of Chd7 (Feng et al., 2017; Whittaker et al., 2017). Using the *Nestin-Reln* mouse line, Whittaker et al. (2017) show that overexpression of *reelin* during brain development partially rescues defects of *Chd7* mutant cerebellum. Using RNA-seq and ChIP-seq (Chromatin immunoprecipitation with high throughput sequencing) analysis in differentiating OLs, CHD7 was found to regulate genes like *Osterx* and *Creb3l2*, both of them are required for both OL differentiation and bone formation (He et al., 2016). Except the *Otx2* gene, CHD7 functions as a transcriptional activators for its target genes. Genome-wide analysis including RNA-seq and ChIP-seq in GNPs and OLs support the notion that CHD7 is required for the activation of gene expression during neural differentiation (He et al., 2016; Feng et al., 2017).

**Table 2:** Key target genes of CHD7 in mouse neural cells.

Target genes	Neural cells	Direct binding of CHD7	Rescue	Reference
*Jag1*, Gli2, Gli3, *MycN, Hes5*	Neural stem cells (NSCs)	Promoter and enhancer, ChIP-seq	n.d.	Engelen et al., 2011
*Sox4*, *Sox11*	Adult NSCs	Promoter, ChIP-qPCR	Yes	Feng et al., 2013
*Rarb, Rxrg, Neurod1*	Subventricular zone (SVZ) NSCs	Promoter, ChIP-qPCR	Yes	Micucci et al., 2014
*Hes5*	Subgranular zone (SGZ) NSCs; NSCs	Promoter, ChIP-seq	Yes	Engelen et al., 2011; Jones et al., 2015
*Osterix, Creb3l2*	Oligodendrocytes	Promoter and enhancer, ChIP-seq	Yes	He et al., 2016
*Otx2*, *Gbx2*	Cells in rhombomere 1	Enhancers, ChIP-qPCR	n.d.	Yu et al., 2013
*Reelin*	Cerebellar GNPs	Enhancers, ChIP-seq	Yes	Feng et al., 2017; Whittaker et al., 2017


## The Molecular Function of CHD7 in Neural Cells

Data from several CHD7 ChIP-seq studies have shown that CHD7 protein preferentially binds to distal regulatory elements in different cells including ESCs (embryonic stem cells), NPCs, and GNPs (Schnetz et al., 2009, 2010; Ram et al., 2011; Feng et al., 2017). In particular, two studies have shown the association of CHD7 to super-enhancers in human ESCs and mouse GNPs (Hnisz et al., 2013; Feng et al., 2017). Given super-enhancers are associated with genes establishing cell identity, these findings implicate important role of CHD7 in cell fate determination. As an example, two super-enhancers are present at the *Chd7* gene in mouse GNPs (Feng et al., 2017), consistent with the important role of CHD7 in these cells. Using ATAC-seq (transposase-Accessible Chromatin with high throughput sequencing), Feng et al. (2017) show loss of *Chd7* leads to specific alteration of open chromatin structure in distal regulatory elements of *Chd7* target genes. How CHD7 is recruited to specific targets is still an open question. Given CHD7 protein does not have DNA binding specificity, it is believed that sequence-specific transcription factors recruit CHD7 to its target genes. Along with this line, several transcription factors have been reported to interact with CHD7. In mouse NSCs, knock down of *Sox2* impairs the binding of CHD7 to its target, indicating that Sox2 is involved in the recruitment of Chd7 (Engelen et al., 2011). In differentiating OLs, CHD7 is shown to interact with a SoxE family of transcription factor SOX10, and to colocalize with SOX10 genome-wide (He et al., 2016). The requirement of SOX10 for the targeting of CHD7 in OLs has not been shown in this study, however.

As expected, the chromatin remodeling activity is important for the function of CHD7 during development. Most of *CHD7* mutations in CHARGE syndrome patients result in truncated CHD7 protein that apparently lost its ATPase and chromatin remodeling activities. Importantly, Bouazoune and Kingston (2012) observed that several patients-derived missense *CHD7* mutations lead to reduction or loss of its ATPase and remodeling activity *in vitro*. Intriguingly, CHD7 interacts with other chromatin remodelers. Multiple evidences suggest a possible functional interaction between CHD7 and the BAF (Brg1/Brahma-associated factors) complex. **First**, in human neural crest cells, CHD7 is associated with multiple subunits of the PBAF (Polybromo- and Brg1/Brahma-associated factors) complex (Bajpai et al., 2010). CHD7 and PBAF colocalize to distal regulatory elements of key neural crest transcription factors, and synergistically activate neural crest gene expression (Bajpai et al., 2010). Consistently, in neural crest-derived melanocyte, Brg1-CHD7-containing PBAF complex interacts with and facilitates the function of the master transcription factor MITF (Microphthalmia-associated transcription factor) (Laurette et al., 2015). **Second**, in OLs, BRG1 was shown to activate the expression of *Chd7* (He et al., 2016). **Third**, phenotypes of *Brg1* CKO brain in adult neurogenesis and cerebellar development (Ninkovic et al., 2013; Moreno et al., 2014) are similar to *Chd7* CKO brain. Moreover, yeast two-hybrid screening has identified CHD8, another CHD family chromatin remodeler, as an interacting partner of CHD7 (Batsukh et al., 2010). The interaction between CHD7 with BRG1 and CHD8 was confirmed in HEK293T cells using coimmunoprecipitation coupled to mass spectrometry (Feng et al., 2017). How chromatin remodelers function together is an open question. One stimulating study show that three chromatin remodelers BRG1, CHD4, and SNF2H colocalize to a substantial portion of sites on chromatin, and the DNA accessibility of many regions requires a combined activity of several remodelers (Morris et al., 2014). It is worthy to investigate the potential cooperative or counteractive functions of CHD7 with BRG1 and CHD8 during brain development.

Another interesting molecular mechanism revealed from a recent study is the cooperative function of CHD7 and DNA topoisomerase TOP2B in transcriptional regulation (Feng et al., 2017). Increasing evidences show the cooperative role of chromatin remodelers and DNA topoisomerases. In mouse ESCs, BAF complex was shown to regulate DNA decatenation during mitosis by recruiting DNA topoisomerase TOP2A (Dykhuizen et al., 2013). One recent study from the same lab demonstrates that TOP2B synergizes with the BAF complex to resolve facultative chromatin to accessible chromatin (Miller et al., 2017). Another recent study shows that BRG1 is required for the recruitment of Topoisomerase 1 in B-cell line (Husain et al., 2016). Importantly, enzymatic activities of TOP1 and TOP2B are absolutely required for the transcription of long genes (>100 kb in gene length) in neurons (King et al., 2013). Many of expressed long genes in neurons are essential neuronal genes, with their dysfunction results in various human neurological disorders. Feng et al. (2017) demonstrate that CHD7 recruits TOP2B to facilitate the transcription of long genes in cerebellar granule neurons, including the *reelin* gene. Consistently, ablation of *Top2b* specifically in mouse forebrain results in a *reelin*-deficient phenotype in cerebral cortex (Lyu and Wang, 2003).

## Future Perspective

One of remaining questions concerning the function of CHD7 in the brain is whether CHD7 is required for the function of mature neurons. As discussed before, the expression of *Chd7* is switched off in most types of mature neurons in the brain during neurogenesis. However, CHD7 is highly expressed in cerebellar granule neurons in adult mouse and human brain (Feng et al., 2017), and some interneurons in the olfactory bulb of adult mouse (Micucci et al., 2014). The selective expression of *Chd7* indicates that it may be involved in the function of these mature neurons. Studying the function of CHD7 in adult brain may help us to understand neuronal behavior abnormality frequent observed in CHARGE syndrome patients.

Several key questions regarding molecular function of CHD7 in the cell remains to be answered. **First,** what is pathogenic mechanisms of *CHD7* missense mutations? Study of missense mutations could provide important insight into the molecular and biochemical function of CHD7 protein. Recent development of structure analysis of chromatin remodelers may provide crucial mechanistic insight of missense mutations of CHD7. For instance, the chromo domain of CHD1 has been shown to be structurally required for the activity of its ATPase activity (Sundaramoorthy et al., 2017). Given the chromo domains in CHD family of chromatin remodelers are conserved, this finding may provide a mechanistic answer for the apparent loss of ATPase activity of one chromo domain mutation CHD7 S834F (Bouazoune and Kingston, 2012). **Second**, what is the exact function of CHD7 at enhancers? It is tempting to speculate that the nucleosome remodeling activity of CHD7 facilitates the transcription activity at enhancers. This hypothesis remains to be experimentally tested. **Third**, does CHD7 functions alone or within a complex? As examples, CHD1 functions as a monomer (Tran et al., 2000; Lusser et al., 2005), in contrast, CHD4 functions within the NuRD (Nucleosome remodeling Deacetylase) complex (Zhang et al., 1998). Solid biochemical assays need to be performed to answer this question.

It is worthy to notice that recent unprecedented development of technology in the field of human cell-based disease modeling. It has already become a routine practice to derive human induced pluripotent stem cells (iPSCs) from patients. The full reservoir of differentiation capacity enables iPSCs as an excellent cell model for disease modeling. Recent development of genome editing tools such as CRISPR-Cas technology has in principle enabled the genome editing at anywhere of the genome in any cell. In particular, genome editing in human iPSCs has enormous application in biomedical research (Hockemeyer and Jaenisch, 2016). Moreover, the recent development of 3D-based human brain organoid culture system has largely improved our ability to model human brain development in tissue culture dish (Lancaster et al., 2013). These state-of-art human cell-based technologies have been applied to model human brain development and neurological disorders. Study the function of CHD7 using this approach is expected to advance our understanding of role of CHD7 in brain development and disease.

## Author Contributions

All authors listed have made a substantial, direct and intellectual contribution to the work, and approved it for publication.

## Conflict of Interest Statement

The authors declare that the research was conducted in the absence of any commercial or financial relationships that could be construed as a potential conflict of interest.

## Footnotes

1 www.chd7.org

## References

[B1] Bajpai R. Chen D. A. Rada-Iglesias A. Zhang J. Xiong Y. Helms J. (2010). CHD7 cooperates with PBAF to control multipotent neural crest formation. *Nature* 463 958–962. 10.1038/nature08733 20130577 PMC2890258

[B2] Balasubramanian R. Choi J. H. Francescatto L. Willer J. Horton E. R. Asimacopoulos E. P. (2014). Functionally compromised CHD7 alleles in patients with isolated GnRH deficiency. *Proc. Natl. Acad. Sci. U.S.A.* 111 17953–17958. 10.1073/pnas.1417438111 25472840 PMC4273325

[B3] Basson M. A. van Ravenswaaij-Arts C. (2015). Functional insights into chromatin remodelling from studies on CHARGE syndrome. *Trends Genet.* 31 600–611. 10.1016/j.tig.2015.05.009 26411921 PMC4604214

[B4] Batsukh T. Pieper L. Koszucka A. M. von Velsen N. Hoyer-Fender S. Elbracht M. (2010). CHD8 interacts with CHD7, a protein which is mutated in CHARGE syndrome. *Hum. Mol. Genet.* 19 2858–2866. 10.1093/hmg/ddq189 20453063

[B5] Becker R. Stiemer B. Neumann L. Entezami M. (2001). Mild ventriculomegaly, mild cerebellar hypoplasia and dysplastic choroid plexus as early prenatal signs of CHARGE association. *Fetal Diagn. Ther.* 16 280–283. 10.1159/000053928 11509849

[B6] Bergman J. E. Bosman E. A. van Ravenswaaij-Arts C. M. Steel K. P. (2010). Study of smell and reproductive organs in a mouse model for CHARGE syndrome. *Eur. J. Hum. Genet.* 18 171–177. 10.1038/ejhg.2009.158 19809474 PMC2987182

[B7] Bergman J. E. Janssen N. Hoefsloot L. H. Jongmans M. C. Hofstra R. M. van Ravenswaaij-Arts C. M. (2011). CHD7 mutations and CHARGE syndrome: the clinical implications of an expanding phenotype. *J. Med. Genet.* 48 334–342. 10.1136/jmg.2010.087106 21378379

[B8] Blustajn J. Kirsch C. F. Panigrahy A. Netchine I. (2008). Olfactory anomalies in CHARGE syndrome: imaging findings of a potential major diagnostic criterion. *AJNR Am. J. Neuroradiol.* 29 1266–1269. 10.3174/ajnr.A1099 18417599 PMC8119129

[B9] Bosman E. A. Penn A. C. Ambrose J. C. Kettleborough R. Stemple D. L. Steel K. P. (2005). Multiple mutations in mouse Chd7 provide models for CHARGE syndrome. *Hum. Mol. Genet.* 14 3463–3476. 10.1093/hmg/ddi375 16207732

[B10] Bouazoune K. Kingston R. E. (2012). Chromatin remodeling by the CHD7 protein is impaired by mutations that cause human developmental disorders. *Proc. Natl. Acad. Sci. U.S.A.* 109 19238–19243. 10.1073/pnas.1213825109 23134727 PMC3511097

[B11] Chalouhi C. Faulcon P. Le Bihan C. Hertz-Pannier L. Bonfils P. Abadie V. (2005). Olfactory evaluation in children: application to the CHARGE syndrome. *Pediatrics* 116 e81–e88. 10.1542/peds.2004-1970 15958661

[B12] Clapier C. R. Cairns B. R. (2009). The biology of chromatin remodeling complexes. *Annu. Rev. Biochem.* 78 273–304. 10.1146/annurev.biochem.77.062706.153223 19355820

[B13] Dykhuizen E. C. Hargreaves D. C. Miller E. L. Cui K. Korshunov A. Kool M. (2013). BAF complexes facilitate decatenation of DNA by topoisomerase IIalpha. *Nature* 497 624–627. 10.1038/nature12146 23698369 PMC3668793

[B14] Engelen E. Akinci U. Bryne J. C. Hou J. Gontan C. Moen M. (2011). Sox2 cooperates with Chd7 to regulate genes that are mutated in human syndromes. *Nat. Genet.* 43 607–611. 10.1038/ng.825 21532573

[B15] Feng W. Kawauchi D. Korkel-Qu H. Deng H. Serger E. Sieber L. (2017). Chd7 is indispensable for mammalian brain development through activation of a neuronal differentiation programme. *Nat. Commun.* 8:14758 10.1038/ncomms14758 PMC5364396 28317875

[B16] Feng W. Khan M. A. Bellvis P. Zhu Z. Bernhardt O. Herold-Mende C. (2013). The chromatin remodeler CHD7 regulates adult neurogenesis via activation of SoxC transcription factors. *Cell Stem Cell* 13 62–72. 10.1016/j.stem.2013.05.002 23827709

[B17] Gage F. H. (2000). Mammalian neural stem cells. *Science* 287 1433–1438. 10.1126/science.287.5457.1433 10688783

[B18] Habib N. Li Y. Heidenreich M. Swiech L. Avraham-Davidi I. Trombetta J. J. (2016). Div-Seq: single-nucleus RNA-Seq reveals dynamics of rare adult newborn neurons. *Science* 353 925–928. 10.1126/science.aad7038 27471252 PMC5480621

[B19] Hale C. L. Niederriter A. N. Green G. E. Martin D. M. (2016). Atypical phenotypes associated with pathogenic CHD7 variants and a proposal for broadening CHARGE syndrome clinical diagnostic criteria. *Am. J. Med. Genet. A* 170A, 344–354. 10.1002/ajmg.a.37435 26590800 PMC5102387

[B20] Hall B. D. (1979). Choanal atresia and associated multiple anomalies. *J. Pediatr.* 95 395–398. 10.1016/S0022-3476(79)80513-2 469662

[B21] He D. Marie C. Zhao C. Kim B. Wang J. Deng Y. (2016). Chd7 cooperates with Sox10 and regulates the onset of CNS myelination and remyelination. *Nat. Neurosci.* 19 678–689. 10.1038/nn.4258 26928066 PMC4846514

[B22] Hittner H. M. Hirsch N. J. Kreh G. M. Rudolph A. J. (1979). Colobomatous microphthalmia, heart disease, hearing loss, and mental retardation–a syndrome. *J. Pediatr. Ophthalmol. Strabismus* 16 122–128. 458518 10.3928/0191-3913-19790301-10

[B23] Hnisz D. Abraham B. J. Lee T. I. Lau A. Saint-Andre V. Sigova A. A. (2013). Super-enhancers in the control of cell identity and disease. *Cell* 155 934–947. 10.1016/j.cell.2013.09.053 24119843 PMC3841062

[B24] Ho L. Crabtree G. R. (2010). Chromatin remodelling during development. *Nature* 463 474–484. 10.1038/nature08911 20110991 PMC3060774

[B25] Hockemeyer D. Jaenisch R. (2016). Induced pluripotent stem cells meet genome editing. *Cell Stem Cell* 18 573–586. 27152442 10.1016/j.stem.2016.04.013 PMC4871596

[B26] Hurd E. A. Capers P. L. Blauwkamp M. N. Adams M. E. Raphael Y. Poucher H. K. (2007). Loss of Chd7 function in gene-trapped reporter mice is embryonic lethal and associated with severe defects in multiple developing tissues. *Mamm. Genome* 18 94–104. 10.1007/s00335-006-0107-6 17334657

[B27] Hurd E. A. Poucher H. K. Cheng K. Raphael Y. Martin D. M. (2010). The ATP-dependent chromatin remodeling enzyme CHD7 regulates pro-neural gene expression and neurogenesis in the inner ear. *Development* 137 3139–3150. 10.1242/dev.047894 20736290 PMC2926962

[B28] Husain A. Begum N. A. Taniguchi T. Taniguchi H. Kobayashi M. Honjo T. (2016). Chromatin remodeller SMARCA4 recruits topoisomerase 1 and suppresses transcription-associated genomic instability. *Nat. Commun.* 7:10549 10.1038/ncomms10549 PMC4742980 26842758

[B29] Jiang X. Zhou Y. Xian L. Chen W. Wu H. Gao X. (2012). The mutation in Chd7 causes misexpression of Bmp4 and developmental defects in telencephalic midline. *Am. J. Pathol.* 181 626–641. 10.1016/j.ajpath.2012.05.006 22658483

[B30] Johansson M. Rastam M. Billstedt E. Danielsson S. Stromland K. Miller M. (2006). Autism spectrum disorders and underlying brain pathology in CHARGE association. *Dev. Med. Child Neurol.* 48 40–50. 10.1017/S0012162206000090 16359593

[B31] Jones K. M. Saric N. Russell J. P. Andoniadou C. L. Scambler P. J. Basson M. A. (2015). CHD7 maintains neural stem cell quiescence and prevents premature stem cell depletion in the adult hippocampus. *Stem Cells* 33 196–210. 10.1002/stem.1822 25183173 PMC5952591

[B32] Kim H. G. Kurth I. Lan F. Meliciani I. Wenzel W. Eom S. H. (2008). Mutations in CHD7, encoding a chromatin-remodeling protein, cause idiopathic hypogonadotropic hypogonadism and Kallmann syndrome. *Am. J. Hum. Genet.* 83 511–519. 10.1016/j.ajhg.2008.09.005 18834967 PMC2561938

[B33] King I. F. Yandava C. N. Mabb A. M. Hsiao J. S. Huang H. S. Pearson B. L. (2013). Topoisomerases facilitate transcription of long genes linked to autism. *Nature* 501 58–62. 10.1038/nature12504 23995680 PMC3767287

[B34] Lancaster M. A. Renner M. Martin C. A. Wenzel D. Bicknell L. S. Hurles M. E. (2013). Cerebral organoids model human brain development and microcephaly. *Nature* 501 373–379. 10.1038/nature12517 23995685 PMC3817409

[B35] Laurette P. Strub T. Koludrovic D. Keime C. Le Gras S. Seberg H. (2015). Transcription factor MITF and remodeller BRG1 define chromatin organisation at regulatory elements in melanoma cells. *Elife* 4:e06857 10.7554/eLife.06857 PMC4407272 25803486

[B36] Layman W. S. Hurd E. A. Martin D. M. (2010). Chromodomain proteins in development: lessons from CHARGE syndrome. *Clin. Genet.* 78 11–20. 10.1111/j.1399-0004.2010.01446.x 20507341 PMC3097394

[B37] Layman W. S. Hurd E. A. Martin D. M. (2011). Reproductive dysfunction and decreased GnRH neurogenesis in a mouse model of CHARGE syndrome. *Hum. Mol. Genet.* 20 3138–3150. 10.1093/hmg/ddr216 21596839 PMC3140819

[B38] Layman W. S. McEwen D. P. Beyer L. A. Lalani S. R. Fernbach S. D. Oh E. (2009). Defects in neural stem cell proliferation and olfaction in Chd7 deficient mice indicate a mechanism for hyposmia in human CHARGE syndrome. *Hum. Mol. Genet.* 18 1909–1923. 10.1093/hmg/ddp112 19279158 PMC2678924

[B39] Legendre M. Gonzales M. Goudefroye G. Bilan F. Parisot P. Perez M. J. (2012). Antenatal spectrum of CHARGE syndrome in 40 fetuses with CHD7 mutations. *J. Med. Genet.* 49 698–707. 10.1136/jmedgenet-2012-100926 23024289

[B40] Lin A. E. Siebert J. R. Graham J. M. Jr. (1990). Central nervous system malformations in the CHARGE association. *Am. J. Med. Genet.* 37 304–310. 10.1002/ajmg.1320370303 2260555

[B41] Lusser A. Urwin D. L. Kadonaga J. T. (2005). Distinct activities of CHD1 and ACF in ATP-dependent chromatin assembly. *Nat. Struct. Mol. Biol.* 12 160–166. 10.1038/nsmb884 15643425

[B42] Lyu Y. L. Wang J. C. (2003). Aberrant lamination in the cerebral cortex of mouse embryos lacking DNA topoisomerase IIbeta. *Proc. Natl. Acad. Sci. U.S.A.* 100 7123–7128. 10.1073/pnas.1232376100 12773624 PMC165840

[B43] Marcos S. Sarfati J. Leroy C. Fouveaut C. Parent P. Metz C. (2014). The prevalence of CHD7 missense versus truncating mutations is higher in patients with Kallmann syndrome than in typical CHARGE patients. *J. Clin. Endocrinol. Metab.* 99 E2138–E2143. 10.1210/jc.2014-2110 25077900

[B44] Martynoga B. Mateo J. L. Zhou B. Andersen J. Achimastou A. Urban N. (2013). Epigenomic enhancer annotation reveals a key role for NFIX in neural stem cell quiescence. *Genes Dev.* 27 1769–1786. 10.1101/gad.216804.113 23964093 PMC3759694

[B45] Melicharek D. J. Ramirez L. C. Singh S. Thompson R. Marenda D. R. (2010). Kismet/CHD7 regulates axon morphology, memory and locomotion in a *Drosophila* model of CHARGE syndrome. *Hum. Mol. Genet.* 19 4253–4264. 10.1093/hmg/ddq348 20716578 PMC2951870

[B46] Micucci J. A. Layman W. S. Hurd E. A. Sperry E. D. Frank S. F. Durham M. A. (2014). CHD7 and retinoic acid signaling cooperate to regulate neural stem cell and inner ear development in mouse models of CHARGE syndrome. *Hum. Mol. Genet.* 23 434–448. 10.1093/hmg/ddt435 24026680 PMC3869363

[B47] Miller E. L. Hargreaves D. C. Kadoch C. Chang C. Y. Calarco J. P. Hodges C. (2017). TOP2 synergizes with BAF chromatin remodeling for both resolution and formation of facultative heterochromatin. *Nat. Struct. Mol. Biol.* 24 344–352. 10.1038/nsmb.3384 28250416 PMC5395302

[B48] Moreno N. Schmidt C. Ahlfeld J. Poschl J. Dittmar S. Pfister S. M. (2014). Loss of Smarc proteins impairs cerebellar development. *J. Neurosci.* 34 13486–13491. 10.1523/JNEUROSCI.2560-14.2014 25274825 PMC6608313

[B49] Morris S. A. Baek S. Sung M. H. John S. Wiench M. Johnson T. A. (2014). Overlapping chromatin-remodeling systems collaborate genome wide at dynamic chromatin transitions. *Nat. Struct. Mol. Biol.* 21 73–81. 10.1038/nsmb.2718 24317492 PMC3947387

[B50] Ninkovic J. Steiner-Mezzadri A. Jawerka M. Akinci U. Masserdotti G. Petricca S. (2013). The BAF complex interacts with Pax6 in adult neural progenitors to establish a neurogenic cross-regulatory transcriptional network. *Cell Stem Cell* 13 403–418. 10.1016/j.stem.2013.07.002 23933087 PMC4098720

[B51] Pagon R. A. Graham J. M. Jr. Zonana J. Yong S. L. (1981). Coloboma, congenital heart disease, and choanal atresia with multiple anomalies: CHARGE association. *J. Pediatr.* 99 223–227. 10.1016/S0022-3476(81)80454-4 6166737

[B52] Patten S. A. Jacobs-McDaniels N. L. Zaouter C. Drapeau P. Albertson R. C. Moldovan F. (2012). Role of Chd7 in zebrafish: a model for CHARGE syndrome. *PLOS ONE* 7:e31650 10.1371/journal.pone.0031650 PMC3282775 22363697

[B53] Pinto G. Abadie V. Mesnage R. Blustajn J. Cabrol S. Amiel J. (2005). CHARGE syndrome includes hypogonadotropic hypogonadism and abnormal olfactory bulb development. *J. Clin. Endocrinol. Metab.* 90 5621–5626. 10.1210/jc.2004-2474 16030162

[B54] Ram O. Goren A. Amit I. Shoresh N. Yosef N. Ernst J. (2011). Combinatorial patterning of chromatin regulators uncovered by genome-wide location analysis in human cells. *Cell* 147 1628–1639. 10.1016/j.cell.2011.09.057 22196736 PMC3312319

[B55] Sanlaville D. Etchevers H. C. Gonzales M. Martinovic J. Clement-Ziza M. Delezoide A. L. (2006). Phenotypic spectrum of CHARGE syndrome in fetuses with CHD7 truncating mutations correlates with expression during human development. *J. Med. Genet.* 43 211–217. 10.1136/jmg.2005.036160 16169932 PMC2563257

[B56] Schnetz M. P. Bartels C. F. Shastri K. Balasubramanian D. Zentner G. E. Balaji R. (2009). Genomic distribution of CHD7 on chromatin tracks H3K4 methylation patterns. *Genome Res.* 19 590–601. 10.1101/gr.086983.108 19251738 PMC2665778

[B57] Schnetz M. P. Handoko L. Akhtar-Zaidi B. Bartels C. F. Pereira C. F. Fisher A. G. (2010). CHD7 targets active gene enhancer elements to modulate ES cell-specific gene expression. *PLOS Genet.* 6:e1001023 10.1371/journal.pgen.1001023 PMC2904778 20657823

[B58] Sundaramoorthy R. Hughes A. L. Singh V. Wiechens N. Ryan D. P. El-Mkami H. (2017). Structural reorganization of the chromatin remodeling enzyme Chd1 upon engagement with nucleosomes. *Elife* 6:e22510 10.7554/eLife.22510 PMC5391205 28332978

[B59] Tellier A. L. Cormier-Daire V. Abadie V. Amiel J. Sigaudy S. Bonnet D. (1998). CHARGE syndrome: report of 47 cases and review. *Am. J. Med. Genet.* 76 402–409. 10.1002/(SICI)1096-8628(19980413)76:5<402::AID-AJMG7>3.0.CO;2-O 9556299

[B60] Tran H. G. Steger D. J. Iyer V. R. Johnson A. D. (2000). The chromo domain protein chd1p from budding yeast is an ATP-dependent chromatin-modifying factor. *EMBO J.* 19 2323–2331. 10.1093/emboj/19.10.2323 10811623 PMC384354

[B61] Van Nostrand J. L. Brady C. A. Jung H. Fuentes D. R. Kozak M. M. Johnson T. M. (2014). Inappropriate p53 activation during development induces features of CHARGE syndrome. *Nature* 514 228–232. 10.1038/nature13585 25119037 PMC4192026

[B62] Vissers L. E. van Ravenswaaij C. M. Admiraal R. Hurst J. A. de Vries B. B. Janssen I. M. (2004). Mutations in a new member of the chromodomain gene family cause CHARGE syndrome. *Nat. Genet.* 36 955–957. 10.1038/ng1407 15300250

[B63] Whittaker D. E. Riegman K. L. Kasah S. Mohan C. Yu T. Sala B. P. (2017). The chromatin remodeling factor CHD7 controls cerebellar development by regulating reelin expression. *J. Clin. Invest.* 127 874–887. 10.1172/JCI83408 28165338 PMC5330721

[B64] Yu T. Meiners L. C. Danielsen K. Wong M. T. Bowler T. Reinberg D. (2013). Deregulated FGF and homeotic gene expression underlies cerebellar vermis hypoplasia in CHARGE syndrome. *Elife* 2:e01305 10.7554/eLife.01305 PMC3870572 24368733

[B65] Zentner G. E. Hurd E. A. Schnetz M. P. Handoko L. Wang C. Wang Z. (2010). CHD7 functions in the nucleolus as a positive regulator of ribosomal RNA biogenesis. *Hum. Mol. Genet.* 19 3491–3501. 10.1093/hmg/ddq265 20591827 PMC2928125

[B66] Zhang Y. LeRoy G. Seelig H. P. Lane W. S. Reinberg D. (1998). The dermatomyositis-specific autoantigen Mi2 is a component of a complex containing histone deacetylase and nucleosome remodeling activities. *Cell* 95 279–289. 9790534 10.1016/s0092-8674(00)81758-4

